# Predicting perineal trauma during childbirth using data from a general obstetric population

**DOI:** 10.12688/hrbopenres.13656.2

**Published:** 2023-10-10

**Authors:** Gillian M. Maher, Laura J. O'Byrne, Joye McKernan, Paul Corcoran, Richard A. Greene, Ali S. Khashan, Fergus P. McCarthy

**Affiliations:** 1 INFANT Research Centre, University College Cork, Cork, T12YE02, Ireland; 2 School of Public Health, University College Cork, Cork, T12XF62, Ireland; 3 Department of Obstetrics and Gynaecology, University College Cork, Cork, T12YE02, Ireland; 4 National Perinatal Epidemiology Centre, University College Cork, Cork, T12YE02, Ireland

**Keywords:** Perineal trauma, Prediction model, Internal validation

## Abstract

**Background:**

Perineal trauma is a common complication of childbirth and can have serious impacts on long-term health. Few studies have examined the combined effect of multiple risk factors. We developed and internally validated a risk prediction model to predict third and fourth degree perineal tears using data from a general obstetric population.

**Methods:**

Risk prediction model using data from all singleton vaginal deliveries at Cork University Maternity Hospital (CUMH), Ireland during 2019 and 2020. Third/fourth degree tears were diagnosed by an obstetrician or midwife at time of birth and defined as tears that extended into the anal sphincter complex or involved both the anal sphincter complex and anorectal mucosa. We used univariable and multivariable logistic regression with backward stepwise selection to develop the models. Candidate predictors included infant sex, maternal age, maternal body mass index, parity, mode of delivery, birthweight, post-term delivery, induction of labour and public/private antenatal care. We used the receiver operating characteristic (ROC) curve C-statistic to assess discrimination, and bootstrapping techniques were used to assess internal validation.

**Results:**

Of 8,403 singleton vaginal deliveries, 8,367 (99.54%) had complete data on predictors for model development. A total of 128 women (1.53%) had a third/fourth degree tear. Three variables remained in the final model: nulliparity, mode of delivery (specifically forceps delivery or ventouse delivery) and increasing birthweight (per 100 gram increase) (C-statistic: 0.75, 95% CI: 0.71, 0.79). We developed a nomogram to calculate individualised risk of third/fourth degree tears using these predictors. Bootstrapping indicated good internal performance.

**Conclusions:**

Use of our nomogram can provide an individualised risk assessment of third/fourth degree tears and potentially aid counselling of women on their potential risk.

## Introduction

Perineal trauma is a very common complication of childbirth, estimated to affect up to 80% of women ^1^. Severity of tears can vary considerably and can be classified into four categories from first to fourth degree. First degree tears involve injury to the perineal skin or vaginal mucosa, second degree tears extend deeper involving perineal muscles; third degree tears extend into the anal sphincter complex, while a fourth degree tears involves both the anal sphincter complex and anorectal mucosa ^2, 3^.

The most common tear is first or second degree tear, occurring in up to ~78% of deliveries ^1^. More severe injuries (third and fourth degree) occur in approximately 5–8% of primiparous women and 2–3% of multiparous women ^1^. This can lead to serious impacts on women’s long-term health, such as long term pelvic floor dysfunction, prolonged pain, sexual dysfunction and faecal incontinence ^2, 4^; the latter occurring in almost 40% of women who sustain third and fourth degree tears, despite efforts of primary repair ^5^.

Several individual risk factors for perineal tears have been identified in the literature, including nulliparity, operative vaginal delivery, high birthweight, gestational age, and foetal head circumference ^1, 2, 6^. However, few studies have examined the combined effect of multiple risk factors. Efforts to predict perineal tears using data available during the antepartum and intrapartum period are warranted in order to inform clinical decision-making, accurately counsel women on their individualized risk and increase patient understanding of the potential long-term consequences of specific medical interventions.

Therefore, given their long-term health impacts, the aim of this study was to develop and validate a risk prediction model to predict third and fourth degree perineal tears, using antepartum and intrapartum data from a general obstetric population.

## Methods

### Study population

A national project called ‘The Maternal and Newborn Clinical Management System (MN-CMS)’ was rolled out in the Republic of Ireland in December 2016 ^7^. With this system, an electronic health record was created resulting in a move from paper clinical notes, allowing for all maternal and newborn information to be stored on one record. The first maternity hospital to implement the electronic health record in the Republic of Ireland was Cork University Maternity Hospital (CUMH). As a result, we used anonymised data from all singleton vaginal deliveries at CUMH from January 2019 to December 2020 to develop and internally validate a risk prediction model for third and fourth degree perineal tears.

We obtained ethical approval from the Clinical Research Ethics Committee of the Cork Teaching Hospitals (CREC) (ECM4(v)09/04/2020) in June 2020. The Transparent Reporting of a multivariable prediction model for Individual Prognosis or Diagnosis (TRIPOD) checklist was used as a guideline for reporting our study ^8^ (available here).

### Candidate predictors and outcome

In order to identify candidate predictors, we reviewed existing literature, used expert opinion (comprising obstetricians, epidemiologists and experts on the MN-CMS), and examined the distribution of the predictor in the data available to us, (for example, we excluded any variables with <5 exposed cases) ^9^ to identify routinely measured candidate predictors for third and fourth degree tears.

Candidate predictors considered for model development included infant sex, maternal age, maternal body mass index (BMI), parity, mode of delivery, birthweight, post-term delivery, induction of labour and public/private antenatal care.

A description of candidate predictors is as follows: *infant sex* was categorised as male/female. *Maternal age:* this was recorded in units of years at the initial prenatal visit. *Maternal BMI:* maternal height (cm) and weight (kg) at initial prenatal visit were used to calculate maternal BMI. This was categorised as underweight <18.5, normal weight ≥18.5 to ≤24.9, overweight ≥25 to ≤29.9 and obese ≥30 (due to small numbers, underweight and normal weight were combined). *Parity:* this was recorded as number of previous completed pregnancies and was re-categorised nulliparous or multiparous. *Mode of delivery* (with manual support technique ^10^) was categorised into four different groups as follows: spontaneous vaginal delivery with episiotomy, spontaneous vaginal delivery without episiotomy, forceps delivery and ventouse delivery. We grouped ventouse delivery (with and without episiotomy) and forceps delivery (with and without episiotomy) together due to the small number of these instrumental deliveries that occurred without episiotomy. An episiotomy was defined as a right mediolateral incision ( *i.e.*, a cut made by the doctor or midwife during childbirth that begins in the middle of the vaginal opening and extends down toward the buttocks at a 45-degree angle). Caesarean deliveries were excluded as only vaginal deliveries are at risk of perineal tears. *Birthweight* was measured to the nearest gram and analysed as an increase in risk per 100 gram increase in birthweight. *Post-term delivery* was defined as delivery at ≥40 weeks’ gestation (with estimated due date confirmed by first trimester ultrasound) compared to delivery at or before full-term ( *i.e.*, delivery at <40 weeks’ gestation). Induction of labour was recorded in the MN-CMS if any of the following methods were administered: artificial rupture of membranes, Dilapan-S®, balloon catheter, or prostaglandin gel. Public antenatal care was defined as free antenatal care through the Maternity and Infant Care Scheme in Ireland. This is available to anyone who lives in Ireland or intends to live there for at least one year. Private antenatal care was defined as choosing to pay a consultant's fee/hospital fee so that a particular obstetrician would provide the care throughout the pregnancy/birth and that recovery would take place in a private/semi-private hospital room.

*Outcome:* Degree of tear was diagnosed by an obstetrician or midwife at time of birth and recorded in the MN-CMS. Third/fourth degree tears were tears that extended into the anal sphincter complex or involved both the anal sphincter complex and anorectal mucosa.

### Statistical analysis

All statistical analyses were performed using Stata MP 14.2 (RRID:SCR_012763) (free alternative, RStudio). We used univariable analysis to examine associations between candidate predictors and third/fourth degree tears. To develop the prediction model, any variables that were statistically significant in the univariable analysis ( *i.e.*, p-value < 0.1) were included in multivariable logistic regression with backward stepwise selection (with a p-value of 0.1 for exclusion). Therefore, all candidate predictors considered statistically significant in the univariable analysis were included at first and the least useful predictors ( *i.e.*, the variable that is the least statistically significant) were subsequently removed one-by-one.

*Sample size calculations:* We used the *pmsampsize* command to calculate the minimum sample size and number of events required for model development to minimise overfitting *.* Assuming an outcome event proportion (prevalence) of 0.015, a c-statistic of 0.75, a target shrinkage factor of 0.9, and 12 predictors/categories, then a minimum sample size of 7,995 (with 120 events) would be required to minimise overfitting ^11^.

*Model performance:* Spline functions with 3, 4 and 5 knots were used to assess non-linear functions for any continuous predictors included in model development. These were plotted against the original variable to compare the linear function with the spline function, while Akaike information criterion (AIC) and Bayesian information criterion (BIC) statistics were calculated to examine model fit.

We examined model performance by assessing overall fit, discrimination and calibration. Brier Score and Cragg & Uhler’s (Nagelkerke) R ^2^ assessed overall fit. The area under the receiver operating characteristic curve (ROC) C-statistic assessed discrimination ( *i.e.*, how well the model differentiates between those patients who experience third/fourth degree tears and those who do not ^9^). Calibration-in-the-large (CITL), calibration slope (C-slope) and calibration plot ( *pmcalplot*) of observed against expected probabilities across 10 risk groups of individuals assessed calibration ( *i.e.*, how closely the predictions of the models match the observed outcomes in the data ^9, 12^).

*Internal validation*: To examine internal validation of our model, assess overfitting and calculate the optimism adjusted C-statistic, CITL and C-slope, we used bootstrapping techniques (with 1,000 repetitions). Finally, a graphical representation of our prediction model ( *i.e.*, nomogram) was developed to provide individualised risk assessment for third/fourth degree tears.

## Results

There was a total of 8,403 singleton vaginal deliveries at CUMH during 2019 and 2020, of which 8,367 (99.54%) had complete data on predictors for model development. A total of 128 women (1.53%) were recorded as sustaining third/fourth degree tears (n=123 and 5 respectively). Mother and child characteristics of study participants who did and did not sustain a third/fourth degree tear are outlined in Table 1. Obstetric characteristics of study participants by parity and results of univariable analysis are shown in Tables A1 and A2 as *Extended data* ^8^, with nulliparity, mode of delivery, increasing birthweight and post-term delivery significantly associated with an increased risk of third/fourth degree tears. These variables were used in the multivariable logistic regression with backward stepwise selection to develop the prediction model for third/fourth degree tears.

**Table 1.** Characteristics of study participants who did and did not sustain a third/fourth degree tear.

Characteristic	Did not sustain third/fourth degree tear N=8239	Sustained third/fourth degree tear N=128
***Infant sex***		
Female	4,099 (49.7)	65 (50.8)
Male	4,140 (50.3)	63 (49.2)
***Maternal age (years)***	33.7 (5.1)	33.2 (4.3)
***Maternal BMI***		
Underweight/normal weight	4,158 (50.5)	67 (52.3)
Overweight	2,455 (29.80)	45 (35.2)
Obese	1,408 (17.1)	14 (10.9)
Unknown	218 (2.6)	2 (1.6)
***Parity***		
≥1	5,098 (61.9)	30 (23.4)
0	3,141 (38.1)	98 (76.6)
***Mode of delivery with/without episiotomy***		
SVD without episiotomy	5,728 (69.5)	53 (41.4)
SVD with episiotomy	577 (7.0)	12 (9.4)
Forceps delivery	409 (5.0)	19 (14.8)
Ventouse delivery	1,525 (18.5)	44 (34.4)
***Birthweight (grams)***	3,452.0 (523.8)	3591.4 (484.0)
***Post-term delivery***		
<40 weeks’ gestation	4,366 (53.0)	56 (43.8)
≥40 weeks’ gestation	3,873 (47.0)	72 (56.2)
***Induction of labour***		
No	6,453 (78.3)	101 (78.9)
Yes	1,786 (21.7)	27 (21.1)
***Public/private antenatal care***		
Private	1,415 (17.2)	16 (12.5)
Public	6,834 (82.8)	112 (87.5)

N (%) for categorical variables, mean (SD) for continuous variables. Abbreviations: BMI, body mass index; SVD, spontaneous vaginal delivery.

### Risk prediction model

*Third/fourth degree tears:* Three variables were considered the best combined predictors of third/fourth degree tears using multivariable logistic regression with backward stepwise selection (C-statistic: 0.75, 95% CI: 0.71, 0.79). These included parity (specifically nulliparous), mode of delivery (specifically forceps delivery or ventouse delivery) and increasing birthweight (per 100 gram increase) ( Table 2).

**Table 2.** Best combined predictors for third/fourth degree tear and assessment of model performance.

Characteristic	Coefficient (95% CI)	N (%) or Mean (SD)	OR (95% CI)
***Parity***			
≥1	-	5,128 (61.3)	ref
0	1.56 (1.11, 2.01)	3,239 (38.7)	4.75 (3.03, 7.44)
***Mode of delivery***			
SVD without episiotomy	-	5,781 (69.1)	ref
Forceps delivery	0.71 (0.16, 1.26)	428 (5.1)	2.03 (1.17, 3.51)
Ventouse delivery	0.40 (-0.02, 0.81)	1,569 (18.8)	1.48 (0.98, 2.24)
***Birthweight ^a^***	0.07 (0.03, 0.10)	3,454.1 (523.4)	1.07 (1.03, 1.11)
**Intercept**	-7.62 (-6.31, -8.92)	-	-
***Discrimination***	**Original apparent**	**Optimism**	**Optimism adjusted**
C-statistic	0.75 (95% CI: 0.71, 0.79)	0.01	0.74
***Calibration***			
CITL	0 (95% CI: -0.17, 0.17)	0.001	-0.001
C-slope	1 (95% CI: 0.79, 1.20)	0.06	0.94

**^a^** Per 100 gram increase in birthweight Abbreviations: SD, standard deviation; OR, odds ratio; 95% CI, 95% confidence interval; ref, reference category; SVD, spontaneous vaginal delivery; CITL, calibration-in-the-large; C-slope, calibration slope.

**Figure 1.**
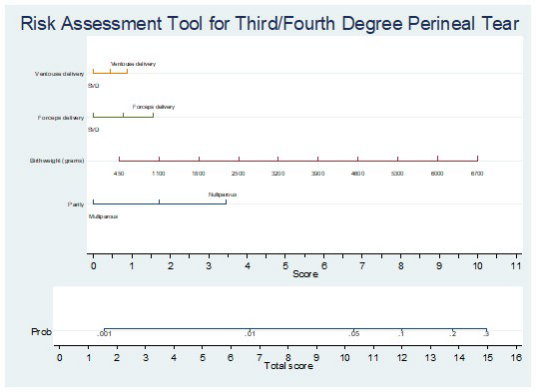
Nomogram providing individualised risk assessment of third/fourth degree perineal tear. For example, a forceps delivery (score 1.5), birthweight of ~4,600 grams (score 7), and nulliparous woman (score 3.5), the total score is 12, corresponding to an ~10% risk of third/fourth degree perineal tear.

We developed a nomogram to provide an individualised risk assessment of third/fourth degree perineal tear using these predictors ( Figure 1). For example, a forceps delivery (score 1.5), birthweight of ~4,600 grams (score 7), and nulliparous woman (score 3.5), the total score is 12, corresponding to an ~10% risk of third/fourth degree perineal tear.

### Model performance and internal validation

There was little difference between the shape of the linear function for birthweight compared to the spline function using 3 and 4 knots, while 5 knots overfit the data (Figure A1, found as *Extended data* ^8^). The AIC and BIC statistics were lowest for the linear function; therefore, birthweight was analysed as a linear function.

The result for the Brier Score and Cragg & Uhler’s (Nagelkerke) R ^2^ were 0.014 and 0.083, respectively. Average model predictions matched average observed outcomes for the 10 risk groups of individuals ( *i.e.*, the deciles of risk that were used as cut-off points to compare observed and expected probabilities in groups of individuals), as indicated by the calibration plot, suggesting good calibration. The majority of the deciles are clustered in the bottom left, suggesting most women have low risk of third/fourth degree tears. There is some miscalibration at the individual level in the higher risk individuals as suggested by the LOWESS smoother. However, there is very little data at the higher risk probabilities as indicated by the spike plot towards the bottom of the graph (Figure A2, found as *Extended data* ^8^).

The original apparent C-statistic was 0.75 (95% CI: 0.71, 0.79). After bootstrapping, there was minimal optimism adjustment to the C-statistic, suggesting good internal performance (optimism adjusted C-statistic: 0.74). The miscalibration in CITL and C-slope were small indicating that overfitting was unlikely to be an issue ( Table 2).

## Discussion

We developed and internally validated a risk prediction model for third/fourth degree perineal tears using antepartum and intrapartum data from a general obstetric Irish population.

During model development, we identified three variables that were considered the best combined predictors of third/fourth degree tears, including nulliparity, mode of delivery (specifically forceps delivery or ventouse delivery) and increasing birthweight. Our model had good internal performance, with an original apparent C-statistic of 0.75, which was minimally adjusted after bootstrapping (optimism adjusted C-statistic: 0.74). Finally, overall calibration of our model was good as suggested by the CITL, C-slope and calibration plot.

Risk prediction models in other geographical locations have been developed using data available before and after delivery, with some similarities to the current study. For example, a single-site model developed in a tertiary hospital in the US (with a ROC curve estimate of 0.83) identified nulliparity, operative vaginal delivery and estimated foetal weight >3,500 grams as risk factors for third/fourth degree tears, while African American ethnicity and tobacco use showed a protective effect ^13^. This study did not differentiate between different types of operative vaginal delivery, however. Separately, a risk stratification tool developed in the US used a scoring system to predict third/fourth degree tears, identifying parity, duration of second stage of labour, vacuum delivery, history of anal sphincter injury, maternal age, gestational age and maternal ethnicity as important risk factors for model development ^14^.

Prediction models using data available before delivery only have also been developed. One Danish model used single-site data available prior to delivery to develop and internally validate a prediction model for obstetric anal sphincter injuries (third-and-fourth-degree tears) ^6^. Variables identified as predictors of third/fourth degree tears (with a C-statistic of 0.71) included suspected macrosomia, nulliparity, increasing maternal age, occiput posterior foetal position and induction/augmentation of labour ^6^. A US-based study developed and validated a prediction model for obstetric anal sphincter injuries using data available at the time of admission for labour only. Out of 30 candidate risk factors identified, 15 remained in the final model. These included parity, maternal age, ethnicity, marital status, insurance status, maternal smoking, gestational age, prior caesarean section, prior operative delivery, anaemia, cardiovascular disease, gestational diabetes, white blood cell and haematocrit values and whether a creatinine lab test was conducted, resulting in a C-statistic of 0.77 ^15^. Although authors had a large number of candidate predictors included in the model, this did not significantly improve model accuracy in comparison to our model. Additionally, we used data available in both the antepartum and intrapartum period to examine any additional potential risk occurring from medical interventions such as mode of delivery (including spontaneous vaginal delivery with and without episiotomy, forceps delivery and ventouse delivery).

### Strengths and limitations

This study contained some limitations that are important to note. First, we did not have access to data on previous history of third/fourth degree tears, length of second stage of labour, birthing position, or indication for instrumental delivery, which may have improved the accuracy of our model. However, before additional candidate predictors are added to a prediction model it is important to consider availability of an appropriate sample size to minimise overfitting. Additionally, regarding a lack of data on previous history of third/fourth degree tears, evidence examining risk of recurrence of third/fourth tears in subsequent pregnancies is inconsistent, and women who had an anal sphincter injury in their first pregnancy are more likely to have a caesarean section in their subsequent pregnancy to avoid a recurrent tear ^16– 19^. Second, to minimise overfitting and maximise the number of events and total sample size for the current study, we grouped third and fourth degree tears together and used data from all singleton vaginal deliveries at CUMH during 2019 and 2020 to develop and internally validate our model. Ideally, we would have used 2020 data to conduct a temporal external validation in order to examine reproducibility of our model. However, despite this limitation, a geographical external validation would still be needed to assess generalisability of our findings. As it is recommended that external validation is carried out by an independent research team, we included the estimates needed to calculate the linear predictor of our model to allow for an independent external validation and objective evaluation of model performance ^20^. Third, previous evidence suggests that third/fourth degree tears may be subject to overdiagnosis potentially as a result of anxiety or fear of missing a diagnosis ^21^. However, the rate of third/fourth degree tear reported in the current study (1.53%) was similar to that of the national estimate for 2019–2020 (1.6%–1.9%), reducing the possibility of misclassification of the outcome ^22^. Fourth, there are many benefits of using secondary data for research purposes, in particular regarding the need for fewer resources. In addition to this, the data used in the current study are real world data and while it has deficits, it reflects outcome in practice. However, a reliance on existing data can be a limitation in terms of data availability, unmeasured variables, and uncertainty around data quality. For example, we did not have access to data on why episiotomies were performed, and while episiotomy was defined according to standard practice at CUMH (i.e., right mediolateral incision), this data could not be validated as we were reliant on secondary data only for the current study. Episiotomies angled at 40–60° are associated with a reduced risk of third and fourth degree tears compared to episiotomies with a more acute angle ^23^. Therefore, a validated measure of episiotomy is necessary to maximise model performance. Finally, this study was limited to singleton deliveries only, therefore results of the prediction model should not be generalised to multiple pregnancies.

There are also several strengths in this study. First, we conducted an internal validation of our model allowing us to assess overfitting and calculate an optimism adjusted C-statistic. Second, the amount of missing data for model development was small (<1%), minimising the likelihood of selection bias driven by missing data. Third, we conducted an appropriate sample size calculation to ensure a sufficiently large sample size in order to minimise overfitting. Finally, we developed a nomogram to graphically represent our prediction model. This provides an individualised risk assessment enabling the user to quickly and easily estimate the probability of sustaining a third/fourth degree tear.

## Conclusions

We developed and internally validated a risk prediction model to predict third/fourth degree perineal tears using antepartum and intrapartum data from a general obstetric population. Three routinely collected variables were considered the best combined predictors, including nulliparity, mode of delivery (specifically forceps delivery or ventouse delivery) and increasing birthweight. Use of our nomogram can provide an individualised risk assessment of third/fourth degree tears and potentially aid counselling of women on their potential risk. However, before a risk prediction model can be applied in clinical practice, an independent external validation is needed to assess reproducibility and an impact study is needed to assess its clinical usefulness.

## Data Availability

The data used in this study are not publicly available due to data protection issues. It is the policy of The National Perinatal Epidemiology Centre (NPEC) that all requests for data for research purposes are first considered by a Data Access Committee. More information on the conditions under which access will be granted and who can request data can be found on their Data Access Committee page ( https://www.ucc.ie/en/npec/dataaccesscommittee/dataaccesscommittee/). The datasets used in this study are from The Maternal and Newborn Clinical Management System (MN-CMS) of the Cork University Maternity Hospital (CUMH) from January 2019 to December 2020 and can be found on the NPEC website at https://www.ucc.ie/en/. Request for data access is available from NPEC by completing their Data Access Request Form and returning it to npec@ucc.ie. Zenodo: Predicting Perineal Trauma during Childbirth using data from a General Obstetric Population. https://doi.org/10.5281/zenodo.8379770 ^8^. This project contains the following extended data: TRIPOD checklist Obstetric characteristics of study participants by parity (Table A1) Univariable analysis (Table A2) Linear function for birthweight plotted against spline function for birthweight (Figure A1) Calibration plot (Figure A2) Data are available under the terms of the Creative Commons Attribution 4.0 International license (CC-BY 4.0).
