# Supplementary material for: Predicting perineal trauma during childbirth using data from a general obstetric population
Source: HRB Open Res. 2023 Oct 10;5:79. Originally published 2022 Dec 1. [Version 2] doi: 10.12688/hrbopenres.13656.2 (PMC10624951; doi:10.12688/hrbopenres.13656.2)
Supplement: Peer review history

# Reviewer report: Reviewer response for version 2

**DOI:** 10.21956/hrbopenres.15117.r36502

**Published:** 2023-11-03

**Authors:** Emilia Rotstein

**Affiliations:** 1 Department of Clinical Science, Intervention and Technology - CLINTEC, Karolinska Institutet, Stockholm, Sweden; 2 Karolinska University Hospital, Stockholm, Sweden

Thank you for your responses to my questions and to your amendments to the manuscript.

Is the work clearly and accurately presented and does it cite the current literature?

Yes

If applicable, is the statistical analysis and its interpretation appropriate?

I cannot comment. A qualified statistician is required.

Are all the source data underlying the results available to ensure full reproducibility?

Yes

Is the study design appropriate and is the work technically sound?

Yes

Are the conclusions drawn adequately supported by the results?

Partly

Are sufficient details of methods and analysis provided to allow replication by others?

Partly

Reviewer Expertise:

Urogynaecology, pelvic floor dysfunction, pelvic floor ultrasound

I confirm that I have read this submission and believe that I have an appropriate level of expertise to confirm that it is of an acceptable scientific standard.

# Reviewer report: Reviewer response for version 1

**DOI:** 10.21956/hrbopenres.14934.r35699

**Published:** 2023-09-01

**Authors:** Emilia Rotstein

**Affiliations:** 1 Department of Clinical Science, Intervention and Technology - CLINTEC, Karolinska Institutet, Stockholm, Sweden; 2 Karolinska University Hospital, Stockholm, Sweden

Thank you for giving me the opportunity to review this interesting study.

 This is a register-based study with the overarching aim of developing and internally validating a risk prediction model of obstetric anal sphincter injuries. A total of 8,367 vaginal deliveries were included with a total frequency of 128 (1.53%) cases of third/fourth degree tear, a number which is quite low in western countries today. As result, nulliparity, mode of delivery (specifically forceps delivery or ventouse delivery) and increasing birthweight (per 100- gram increase) were the important factors.

 The study resulted in a risk prevention model identifying nulliparity, mode of delivery (divided into spontaneous vaginal delivery with or without episiotomy, ventouse or forceps delivery) as risk factors of obstetric trauma. Even though this is not novel information, the resulting nomogram displaying individual risk prediction shows promise.

 I would like to congratulate the authors on a nicely thought-through and executed study, however, I do have some questions for the authors:

- How did you choose the candidate predictors and why? You mention several others in your discussion, and whereas information on previous perineal trauma and e.g. foetal head circumference were not available to you, others such as length of second stage or birthing position may well have been included, as well as indication for instrumental delivery.
- Why were episiotomy rate for ventouse and forceps deliveries not included? The risk reducing effect of episiotomies is still being discussed and as such this would have added valuable information.
- How was the diagnosis made? What was the rationale of not subdividing the degree of tear?
- Was there any information on manual perineal support?
- Are there any indications on how and why the episiotomies were performed?
- I find it quite difficult to navigate Table 1 as the background characteristics and obstetric data are all in the same table. May I suggest at least dividing the obstetric variables into primi- and multiparas? I believe this would facilitate the overview.
- Finally, you mention possible overdiagnosis in your section on limitations even though your frequency falls well within your stated national injury rate but fail to mention the limitations of a registry-based study as far as reliability of data is concerned such as the data not having been collected by the researcher, lacking information on confounders, missing information on data quality to highlight a few. Could you please comment?

Is the work clearly and accurately presented and does it cite the current literature?

Yes

If applicable, is the statistical analysis and its interpretation appropriate?

I cannot comment. A qualified statistician is required.

Are all the source data underlying the results available to ensure full reproducibility?

Yes

Is the study design appropriate and is the work technically sound?

Yes

Are the conclusions drawn adequately supported by the results?

Partly

Are sufficient details of methods and analysis provided to allow replication by others?

Partly

Reviewer Expertise:

Urogynaecology, pelvic floor dysfunction, pelvic floor ultrasound

I confirm that I have read this submission and believe that I have an appropriate level of expertise to confirm that it is of an acceptable scientific standard, however I have significant reservations, as outlined above.

# Response

**Published:** 2023-09-27

**Authors:** Gillian Maher

**Affiliations:** University College Cork, Ireland

Dear Prof Emilia Rotstein,

 We thank you for your helpful review of our manuscript entitled *“Predicting perineal trauma during childbirth using data from a general obstetric population”*.

 Please find below an itemized reply addressing each comment in your Reviewer’s Report.

*1. I would like to congratulate the authors on a nicely thought-through and executed study, however, I do have some questions for the authors: How did you choose the candidate predictors and why? You mention several others in your discussion, and whereas information on previous perineal trauma and e.g. foetal head circumference were not available to you, others such as length of second stage or birthing position may well have been included, as well as indication for instrumental delivery.*

               We thank you for your positive comment regarding our study.

 When choosing candidate predictors, we were reliant on what data was available to us in the Maternal and Newborn Clinical Management System (MN-CMS). We chose candidate predictors by reviewing existing literature and through discussion with obstetricians, epidemiologists, and experts on the MN-CMS. We also examined the distribution of the candidate predictor in the data (for example, we excluded any variables with <5 exposed cases).

              

 This is outlined in our Methods as follows:

 “In order to identify candidate predictors, we reviewed existing literature, used expert opinion (comprising obstetricians, epidemiologists and experts on the MN-CMS), and examined the distribution of the predictor in the data available to us, (for example, we excluded any variables with <5 exposed cases) to identify routinely measured candidate predictors for third and fourth degree tears.”

 With regards to your second comment, we agree that access to variables such as length of second stage of labour, birthing position, and indication for instrumental delivery may have improved the accuracy of our model. We have now acknowledged this in our Discussion as follows:

 “We did not have access to data on previous history of third/fourth degree tears, length of second stage of labour, birthing position, or indication for instrumental delivery, which may have improved the accuracy of our model. However, before additional candidate predictors are added to a prediction model it is important to consider availability of an appropriate sample size to minimise overfitting.”

*2. Why were episiotomy rate for ventouse and forceps deliveries not included? The risk reducing effect of episiotomies is still being discussed and as such this would have added valuable information.*

**We grouped ventouse delivery (with and without episiotomy) and forceps delivery (with and without episiotomy) together due to the small number of these instrumental deliveries that occurred without episiotomy. For example, there was only 1 case of perineal trauma for forceps delivery without episiotomy.

 We have now added this information to our Methods as follows:

  “Mode of delivery (with manual support technique(1)) was categorised into four different groups as follows: spontaneous vaginal delivery with episiotomy, spontaneous vaginal delivery without episiotomy, forceps delivery and ventouse delivery. We grouped ventouse delivery (with and without episiotomy) and forceps delivery (with and without episiotomy) together due to the small number of these instrumental deliveries that occurred without episiotomy.”

*3. How was the diagnosis made? What was the rationale of not subdividing the degree of tear?*

               Degree of tear was diagnosed by an obstetrician or midwife at time of birth and recorded in the MN-CMS. Third/fourth degree tears were tears that extended into the anal sphincter complex or involved both the anal sphincter complex and anorectal mucosa. We have outlined this information in our Methods.

 Regarding your second point, we grouped third- and fourth-degree tears together (n=128) to ensure an appropriate number of events in our data so as to minimise overfitting. This decision was made upon completion of our sample size calculation, as outlined in our Methods as follows:

 “ *Sample size calculations:* We used the pmsampsize command to calculate the minimum sample size and number of events required for model development to minimise overfitting. Assuming an outcome event proportion (prevalence) of 0.015, a c-statistic of 0.75, a target shrinkage factor of 0.9, and 12 predictors/categories, then a minimum sample size of 7,995 (with 120 events) would be required to minimise overfitting.”

 We have now acknowledged the limitation of not subdividing the degree of tear in our Discussion as follows:

 “To minimise overfitting and maximise the number of events and total sample size for the current study, we grouped third and fourth degree tears together and used data from all singleton vaginal deliveries at CUMH during 2019 and 2020 to develop and internally validate our model.”

*4. Was there any information on manual perineal support?*

**As outlined to Reviewer 1 (point 2, above), standard practice at CUMH is to use manual support technique for vaginal deliveries. We have incorporated this into our manuscript as follows and referenced the Hals et al study which describes perineal support in detail:

*“*Mode of delivery (with manual support technique (1)) was categorised into four different groups as follows: spontaneous vaginal delivery with episiotomy, spontaneous vaginal delivery without episiotomy, forceps delivery and ventouse delivery.”

*5. Are there any indications on how and why the episiotomies were performed?*

               We do have information on *how* episiotomies were performed – “Episiotomies were performed using a right mediolateral incision (i.e., a cut made by the doctor or midwife during childbirth that begins in the middle of the vaginal opening and extends down toward the buttocks at a 45-degree angle).” This information is outlined in our Methods.

 Conversely, we do not have access to data on *why* episiotomies were performed. We have included this in our Discussion and acknowledged the limitation that data on episiotomy was not validated (as outlined to Reviewer 1 in point 1, above):

 “A reliance on existing data can be a limitation in terms of data availability, unmeasured variables, and uncertainty around data quality. For example, we did not have access to data on why episiotomies were performed, and while episiotomy was defined according to standard practice at CUMH (i.e., right mediolateral incision), this data could not be validated as we were reliant on secondary data only for the current study. Episiotomies angled at 40–60° are associated with a reduced risk of third and fourth degree tears compared to episiotomies with a more acute angle (2) *. *Therefore, a validated measure of episiotomy is necessary to maximise model performance.”

*6. I find it quite difficult to navigate Table 1 as the background characteristics and obstetric data are all in the same table. May I suggest at least dividing the obstetric variables into primi- and multiparas? I believe this would facilitate the overview.*

               Apologies for this – In table 1 we have outlined the characteristics of our study participants according to those who did and did not sustain a third/fourth degree tear. We are very limited in how we can further subdivide this data due to data protection issues (i.e., subdividing by primiparous and multiparous will result in very small cell counts for those who sustained a third/fourth degree tear). Taking mode of delivery as an example, further subdividing our table by parity will result in <10 exposed cases for SVD with episiotomy, forceps, and ventouse delivery among multiparous women. Similarly, it would result in <10 exposed cases for induction of labour and private antenatal care among multiparous women.

 However, to facilitate a clearer overview of our participants, we have now created an additional table in our extended data (Table A1: https://doi.org/10.5281/zenodo.8379770) outlining our obstetric data by parity.

*7. Finally, you mention possible overdiagnosis in your section on limitations even though your frequency falls well within your stated national injury rate but fail to mention the limitations of a registry-based study as far as reliability of data is concerned such as the data not having been collected by the researcher, lacking information on confounders, missing information on data quality to highlight a few. Could you please comment?*

               There are many benefits of using secondary data for research purposes, in particular regarding the need for fewer resources. Furthermore, the data used in the current study are real world data and while it has deficits, it reflects outcome in practice. However, we agree that a reliance on existing data can be a limitation in terms of data availability, unmeasured variables, and uncertainty around data quality.

 We have edited our Discussion and incorporated this into our manuscript as follows:

 “There are many benefits of using secondary data for research purposes, in particular regarding the need for fewer resources. In addition to this, the data used in the current study are real world data and while it has deficits, it reflects outcome in practice. However, a reliance on existing data can be a limitation in terms of data availability, unmeasured variables, and uncertainty around data quality.”

 We hope these amendments address the suggestions raised. Should any further amendments be necessary, we would be happy to address them accordingly. 

 Sincerely,

 Dr Gillian Maher, Dr Laura O’Byrne, Dr Joye McKernan, Dr Paul Corcoran, Prof Richard  Greene, Dr Ali Khashan and Dr Fergus McCarthy

**References**

 1.        Hals E, Øian P, Pirhonen T, Gissler M, Hjelle S, Nilsen EB, et al. A Multicenter Interventional Program to Reduce the Incidence of Anal Sphincter Tears. Obstetrics & Gynecology. 2010;116(4):901-8.

 2.        Stedenfeldt M, Pirhonen J, Blix E, Wilsgaard T, Vonen B, Øian P. Episiotomy characteristics and risks for obstetric anal sphincter injuries: a case-control study. BJOG : an international journal of obstetrics and gynaecology. 2012;119(6):724-30.

# Reviewer report: Reviewer response for version 1

**DOI:** 10.21956/hrbopenres.14934.r35280

**Published:** 2023-08-02

**Authors:** Jouko Pirhonen

**Affiliations:** 1 University Hospital of North Norway, Tromsø, Norway

In this manuscript from Ireland the authors developed and internally validated a risk prediction model to predict third- and fourth-degree perineal tears using data from a general obstetric population. They used univariable and multivariable logistic regression with backward stepwise selection to develop the models. Totally 8,367 cases (singleton vaginal deliveries) completed data on predictors for model development. A total frequency of 128 women (1.53%) had a third/fourth degree tear, the number which is quite low in western countries today. As result, nulliparity, mode of delivery (specifically forceps delivery or ventouse delivery) and increasing birthweight (per 100-gram increase) were the important factors.

 All the risk factors studied in the manuscript are well known. Secondly, similar articles have been published earlier. However, the manuscript has a potential interest. Further, the modern statistics improves the quality of this study.

 The greatest limitation of this register study is the database used. For example, the authors describe the used episiotomy (mediolateral) nicely. However, an information if the episiotomy was cut correctly is missing. The authors could read the study from Stedenfeldt et al. Episiotomy characteristics and risks for obstetric anal sphincter injuries: a case-control study. BJOG 2012;119:724–73 for their future research. Further, there is no mention if manual support technique was used or not. This affects greatly for the risk factors as well for OASIS rate. Please, read the article from Hals et a. A Multicenter Interventional Program to Reduce the Incidence of Anal Sphincter Tears Obstet Gynecol 2010;116:901–8 for future research. And finally, which kind of support technique was used.

Is the work clearly and accurately presented and does it cite the current literature?

Yes

If applicable, is the statistical analysis and its interpretation appropriate?

Yes

Are all the source data underlying the results available to ensure full reproducibility?

Partly

Is the study design appropriate and is the work technically sound?

Yes

Are the conclusions drawn adequately supported by the results?

Yes

Are sufficient details of methods and analysis provided to allow replication by others?

Yes

Reviewer Expertise:

Fetal medicine. Ultrasound. Prevention of serious perineal tears.

I confirm that I have read this submission and believe that I have an appropriate level of expertise to confirm that it is of an acceptable scientific standard.

## References

[1] : Episiotomy characteristics and risks for obstetric anal sphincter injuries: a case-control study. *BJOG* .2012;119(6) : 10.1111/j.1471-0528.2012.03293.x 724-30 10.1111/j.1471-0528.2012.03293.x 22390647PMC3489037

[2] : A multicenter interventional program to reduce the incidence of anal sphincter tears. *Obstet Gynecol* .2010;116(4) : 10.1097/AOG.0b013e3181eda77a 901-908 10.1097/AOG.0b013e3181eda77a 20859154

# Response

**Published:** 2023-09-27

**Authors:** Gillian Maher

**Affiliations:** University College Cork, Ireland

Dear Prof Jouko Pirhonen,

 We thank you for your helpful review of our manuscript entitled *“Predicting perineal trauma during childbirth using data from a general obstetric population”*.

 Please find below an itemized reply addressing each comment in your Reviewer’s Report.

*1.* *The greatest limitation of this register study is the database used. For example, the authors describe the used episiotomy (mediolateral) nicely. However, an information if the episiotomy was cut correctly is missing. The authors could read the study from Stedenfeldt et al. Episiotomy characteristics and risks for obstetric anal sphincter injuries: a case-control study. BJOG 2012;119:724–73 for their future research.*

               We thank you for your positive comment regarding the description of episiotomy in our study and for drawing our attention to the Stedenfeldt et al study. As we have outlined in our manuscript, episiotomy was defined as a right mediolateral incision (i.e., a cut made by the doctor or midwife during childbirth that begins in the middle of the vaginal opening and extends down toward the buttocks at a 45-degree angle). However, we agree that it is a limitation that data on episiotomy was not validated.  

 We have edited our Discussion to acknowledge this limitation and referenced the Stedenfeldt et al study as follows:

 “While episiotomy was defined according to standard practice at CUMH (i.e., right mediolateral incision), this data could not be validated as we were reliant on secondary data only for the current study. Episiotomies angled at 40–60° are associated with a reduced risk of third and fourth degree tears compared to episiotomies with a more acute angle (1). Therefore, a validated measure of episiotomy is necessary to maximise model performance.”

 2. *Further, there is no mention if manual support technique was used or not. This affects greatly for the risk factors as well for OASIS rate. Please, read the article from Hals et a. A Multicenter Interventional Program to Reduce the Incidence of Anal Sphincter Tears Obstet Gynecol 2010;116:901–8 for future research. And finally, which kind of support technique was used.*

            Thank you for bringing the Hals et al study to our attention. Standard practice at CUMH is to use manual support technique for vaginal deliveries. We have incorporated this into our manuscript as follows and referenced the Hals et al study:

 Mode of delivery (with manual support technique (2)) was categorised into four different groups as follows: spontaneous vaginal delivery with episiotomy, spontaneous vaginal delivery without episiotomy, forceps delivery and ventouse delivery.”

 We hope these amendments address the suggestions raised. Should any further amendments be necessary, we would be happy to address them accordingly. 

 Sincerely,

 Dr Gillian Maher, Dr Laura O’Byrne, Dr Joye McKernan, Dr Paul Corcoran, Prof Richard  Greene, Dr Ali Khashan and Dr Fergus McCarthy

**References**

 1.        Stedenfeldt M, Pirhonen J, Blix E, Wilsgaard T, Vonen B, Øian P. Episiotomy characteristics and risks for obstetric anal sphincter injuries: a case-control study. BJOG : an international journal of obstetrics and gynaecology. 2012;119(6):724-30.

 2.        Hals E, Øian P, Pirhonen T, Gissler M, Hjelle S, Nilsen EB, et al. A Multicenter Interventional Program to Reduce the Incidence of Anal Sphincter Tears. Obstetrics & Gynecology. 2010;116(4):901-8.
